# Self-reported asthma and allergies in top athletes compared to the general population - results of the German part of the GA^2^LEN-Olympic study 2008

**DOI:** 10.1186/1710-1492-6-31

**Published:** 2010-11-30

**Authors:** Silke Thomas, Bernd Wolfarth, Caroline Wittmer, Dennis Nowak, Katja Radon

**Affiliations:** 1 Unit for Occupational and Environmental Epidemiology & NetTeaching, Institute and Outpatient Clinic for Occupational, Social and Environmental Medicine, Clinical Center of the Ludwig Maximilian University, Ziemssenstr. 1, 80336 Munich, Germany; 2 Department of Preventive and Rehabilitative Sports Medicine, Technical University Munich, Connollystr. 32, 80809 Munich, Germany

## Abstract

**Background:**

Prevalence of asthma and allergies in top athletes is high. However, most previous studies did not include a general population comparison group. We aimed to compare the prevalence of asthma, allergies and medical treatment in different groups of German top athletes to the general population.

**Methods:**

Prior to the 2008 Summer Olympic Games, 291 German candidates for participation (65%) completed a questionnaire on respiratory and allergic symptoms. Results were compared to those of a general population study in Germany (n = 2425, response 68%). Furthermore, associations between types of sports and the self-reported outcomes were calculated. All models were adjusted for age, sex, level of education and smoking.

**Results:**

Athletes reported significantly more doctors' diagnosed asthma (17% vs. 7%), more current use of asthma medication (10% vs. 4%) and allergic rhinitis (25% vs. 17%) compared to the general population. After adjustment, top athletes only had an increased Odds Ratio for doctor's diagnosed asthma (OR: 1.6; 95% CI 1.1-2.5). Compared to the general population, athletes in endurance sports had an increased OR for doctor's diagnosed asthma (2.4; 1.5-3.8) and current use of asthma medication (1.8; 1.0-3.4). In this group, current wheeze was increased when use of asthma medication was taken into account (1.8; 1.1-2.8). For other groups of athletes, no significantly increased ORs were observed.

**Conclusions:**

Compared to the general population, an increased risk of asthma diagnosis and treatment was shown for athletes involved in endurance sports. This might be due to a better medical surveillance and treatment of these athletes.

## Background

Allergies and asthma are frequent in elite athletes. Previous studies have shown that the prevalence of allergic rhinitis in elite athletes is between 15% and 29% [1-5] and that wheezing is reported by 6% to 15% of athletes [2,3,6]. The prevalence of asthma in athletes was reported to be different, based on the methods used and the athletes included in the study. E.g., in the US, the prevalence differed from 12% among athletes (football players) in 1984 to 15% among athletes participating in the 1996 Olympic Summer Games and up to 22% among athletes who participated in the 1998 Olympic Winter Games [2,7,8]. In Australia the prevalence of diagnosed asthma in Olympic athletes rose from 10% in 1976 up to 21% in 2000 [9]. A five-year follow-up in Finnish swimmers showed that prevalence of current asthma increased from 31% at baseline to 44% at follow-up [10]. Overall, between 7-18% of top athletes seem to use asthma medication [4,7,11-13].

In comparison to non-athletes (medical students, volunteers, general population sample), some earlier studies have observed a higher prevalence of asthma and allergies in top athletes [3,6,13-16]. However, a recent Australian study could not show such differences between the prevalence of asthma among athletes and the general population [12].

Results of previous studies in top athletes indicated that the prevalence of asthma is associated with specific types of sport. Self-reported and doctors diagnosed asthma was most common in athletes in endurance sports like swimming, cycling and cross-country skiing [2,5,6,12-14,16-18]. Furthermore, some studies observed that medical treatment for asthma is also higher in athletes in endurance sports [5,11,13]. In comparison to other athletes, those performing in endurance sports might be at higher risk as they inhale a large amount of allergens and irritants (e.g. swimmers are exposed to chlorine and chloramine) and because the ventilation is increased for a longer period of time [15,19,20].

The knowledge about asthma and allergies in top athletes is essential as the diseases influencing the performance of the athletes. Furthermore, therapy during training and competition has to be optimised. However, some asthma medications are bounded to the anti-doping regulations. Therefore, athletes who need to use inhaled β2-agonists (e.g. Formoterol, Terbutalin) have to be diagnosed with current asthma using standardized protocols to obtain a therapeutic use exemption (TUE) from the grating anti-doping Organizations (e.g. National-Anti-Doping-Organization, International Sport Federation, International Olympic Committee) [21-23].

So far, no data on German top athletes are available. Therefore, one aim of the study was to assess the prevalence of allergic and respiratory diseases and information about medical treatment in German top athletes. Furthermore, we compared the prevalence of self-reported asthma symptoms, allergies and medical treatment in German top athletes to the general population in Germany. In addition, we estimated the associations between types of sports and level of endurance and the self-reported outcomes. These results should help to define which groups of German athletes are at increased risk for asthma and allergies and to provide some insights in the quality of asthma surveillance in athletes and non-athletes.

## Methods

### Study design and participants

Within the framework of the Global Asthma and Allergy European Networks (GA^2^LEN) [24], a cross sectional study was conducted in several European countries to examine the prevalence of asthma and allergies in participants of the Olympic Summer Games in Beijing. Eligible participants were asked to complete the Allergy Questionnaire for Athletes (AQUA) [25], a questionnaire for screening asthma and allergies in top athletes.

Potential candidates were contacted by the German Olympic Sports Federation between March to June 2008 prior to the beginning of the Olympic Games in Beijing. All those eligible received a letter explaining the study, a written informed consent form and the questionnaire. Overall, 291 of about 450 German top athletes completed the questionnaire (response 65%).

The study was approved by the Ethics Committee of the Medical Faculty of the Ludwig-Maximilians-University Munich (071/08).

### General population study

The participants of the general population were recruited within the German Lower Saxony Lung Study (NiLS), a survey that was done in 2002-2004 [26]. Over ten thousand inhabitants (age 18 to 44 years) of four rural towns were invited to answer a mail-in questionnaire. Those without occupational or private farm animal contact (n = 2425, response 68%) were considered as representative general population comparison group for the athletes.

### Study instruments

To allow a comparison to the group of the general population in Germany, besides the AQUA questionnaire [25] (top athletes only), additional questions from the ECHRS (European Community Respiratory Health Survey) questionnaire [27] were used in both studies. These validated questions included symptoms of asthma, allergies and medical treatment as well as socio-demographic data.

Based on the questionnaire results, the following outcomes were defined:

▪ **Current wheeze**: Have you had wheezing or whistling in your chest at any time in the last 12 months?

▪ **Doctor's diagnosed asthma**: Have you ever had asthma? AND Was this confirmed by a doctor?

▪ **Current use of asthma medication**: Are you currently taking any medicines including inhalers, aerosols or tablets for asthma?

▪ **Allergic rhinitis**: Do you have any nasal allergies, including hay fever?

▪ **Current wheeze or use of asthma medication**: In order to assess whether control of asthma symptoms might be better in asthmatic athletes than in the general population, current wheeze and current use of asthma medication were combined as an additional outcome.

To investigate if the prevalence of asthma, allergies and medical treatment differ between different sport categories, type of sport was classified in accordance to Alaranta et al. [13] into one of the following groups:

▪ Endurance sport: water sports, track and field athletics, canoeing, cycling, rowing

▪ Team Sports: basketball, hockey, football, handball, volleyball

▪ Motor Skills Sports: archery, gymnastics, shooting

▪ Others: Badminton, riding, fencing, judo, karate, sailing, table tennis, taekwondo, wrestling

As level of endurance might have an own impact on respiratory symptoms, we additionally classified the athletes into three groups (classification by two medical experts: B.W., F.E.):

▪ low/middle level of endurance: riding, gymnastics, taekwondo, table tennis, shooting, archery, sailing

▪ high level of endurance: basketball, hockey, football, handball, volleyball, fencing, judo, karate, wrestling,

▪ very high level of endurance: water sports, track and field athletics, canoeing, cycling, rowing, throwing,

### Potential confounders

Age, sex, level of education and smoking as potential confounding variables were dichotomised into:

▪ Smoking: never vs. ever

▪ Level of education: < 12 years of education vs. ≥12 years of schooling,

▪ Age: mean of distribution (< vs. ≥32 years).

### Statistical analysis

Chi^2^-tests were used to assess bivariate associations. Multivariate analyses were done using logistic regression models. We used three different exposure variables for the models. The first model investigated differences of the outcome variables between top athletes and the general population. In the second analysis, the prevalence of self-reported outcomes were compared from different sport groups as defined above. The last model classified the athletes according to their endurance level.

All models used the general population as reference category adjusting for age, sex, level of education, and smoking.

Statistical analyses were carried out using SAS (SAS version 9.1; SAS Institute Inc., Cary, NC, USA).

## Results

### Descriptives

In comparison to the general population top athletes were more likely to have a higher level of education (80% vs. 25%), to be under the age of 32 years (85% vs. 46%) and never have smoked (86% vs. 41%) than the general population (table 1).

**Table 1:** Prevalence of allergic rhinitis, respiratory symptoms and asthma treatment by population group and sports category

Variable N (%)	General population	Top athletes	Endurance	Motor Skills	Team Sports	Others
	**N = 2425**	**N = 291**	**N = 140**	**N = 28**	**N = 72**	**N = 51**

Age (< 32 years)	1110 (45.8)	248 (85.2)*	122 (85.7)	18 (64.3)	67 (93.1)	40 (78.4)

Education (> = 12 years of schooling)	608 (25.1)	232 (79.7)*	112 (80.1)	17 (60.7)	63 (87.5)	39 (76.5)

Smoking (never)	1001 (41.3)	247 (86.1)*	126 (92.0)	19 (67.9)	63 (90.0)	38 (74.5)

Allergic rhinitis (ever)	409 (16.9)	71 (25.4)*	34 (24.3)	4 (14.2)	18 (25.0)	15 (29.4)

Current wheeze	322 (13.3)	34 (12.0)	23 (16.4)	1 (3.6)	5 (6.9)	4 (7.8)

Doctor's diagnosed asthma (ever)	174 (7.2)	47 (16.6)*	30 (21.4)	2 (7.1)	9 (12.5)	5 (9.8)

Current use of asthma medication	94 (3.9)	27 (9.5)*	17 (12.1)	1 (3.6)	8 (11.1)	3 (5.9)

Current wheeze or use of asthma medication	348 (14.4)	44 (15.6)	29 (20.7)	2 (7.1)	6 (8.3)	4 (7.8)

*p_Chi²_< 0.05

Dividing the athletes into different sport categories, most of the athletes were involved in endurance sport (48%) followed by team sports (25%). Only 10% did sports requiring motor skills. The majority of the athletes were classified as having a very high (46%) or high (40%) endurance level.

### Prevalence of asthma, allergies and medical treatment

Regarding asthma, allergies and medical treatment, top athletes were more likely to report doctor's diagnosed asthma (17% vs. 7%), use of asthma medication (10% vs. 4%) and hay fever (25% vs. 17%) than the participants of the general population (table 1). No statistically significant difference was seen in the prevalence of asthma symptoms.

Regarding the different sport categories, those in endurance sports had the highest prevalence of diagnosed asthma (21%), followed by team sports (13%). Medical treatment against asthma (12%), wheezing (16%) and current wheezing or use of asthma medication (21%) was also most frequent in endurance athletes. Only hay fever was highest in other sports (29%) followed by team sports (25%) (table 1).

Athletes with a very high level of endurance showed the highest prevalence for all observed outcomes (data not shown).

### Logistic regression models: top athletes vs. general population

After adjusting, a statistically significantly increased risk for doctor's diagnosed of asthma (OR 1.6; 95% CI 1.1-2.5) was seen for the top athletes. Regarding the other outcomes, no statistically significant differences were found between top athletes and the general population (table 2).

**Table 2:** Adjusted Odds Ratios with 95% Confidence Interval for respiratory symptoms and diseases among top athletes.

	Top athletes
	OR (95% CI)*

Allergic rhinitis (ever)	1.1 (0.8-1.5)

Current wheeze	0.9 (0.6-1.4)

Doctor's diagnosed asthma	**1.6 (1.1-2.5)**

Current use of asthma medication	1.4 (0.8-2.3)

Current wheeze or use of asthma medication	1.1 (0.8-1.6)

Reference group: general population (OR = 1)

OR: Odds Ration; CI: Confidence Interval

* adjusted for age, sex, level of education and smoking

### Logistic regression models: Sports categories and endurance level

Dividing the top athletes into different sport categories, those in endurance sports had a statistically significantly increased Odds Ratio for doctor's diagnosed asthma (2.4; 1.5-3.8), current use of asthma medication (1.8; 1.0-3.4) and current wheeze or use of asthma medication (1.8; 1.1-2.8) compared to the general population (table 3). In contrast, no differences were shown with respect to asthma symptoms or allergic rhinitis.

**Table 3:** Adjusted logistic regression models (Odds Ratio with 95%-Confidence Interval) for the association between different sport and endurance categories and self-reported outcomes.

Variable	Sports category [12]				Level of Endurance		
	**Motor Skills** **(n = 28)**	**Team Sports** **(n = 72)**	**Endurance** **(n = 140)**	**Others** **(n = 51)**	**Low/middle** **(n = 42)**	**High** **(n = 115)**	**Very high** **(n = 134)**

Allergic rhinitis (ever)	0.6 (0.2-1.8)	1.2 (0.7-2.1)	1.0 (0.6-1.5)	1.5 (0.8-2.8)	0.9 (0.4-2.0)	1.2 (0.8-2.0)	1.0 (0.6-1.6)

Current wheeze	0.3 (0.0-1.9)	0.6 (0.2-1.6)	1.5 (0.9-2.4)	0.6 (0.2-1.6)	0.3 (0.1-1.4)	0.6 (0.3-1.2)	1.6 (0.9-2.6)

Doctor's diagnosed asthma	0.8 (0.2-3.3)	1.2 (0.5-2.6)	**2.4 (1.5-3.8)**	1.0 (0.4-2.6)	0.8 (0.3-2.8)	1.1 (0.5-2.0)	**2.5 (1.5-4.0)**

Current use of asthma medication	0.6 (0.1-1.8)	1.1 (0.4-2.8)	**1.8 (1.0-3.4)**	0.9 (0.3-3.1)	0.4 (0.1-3.3)	1.1 (0.5-2.3)	**1.9 (1.0-3.5)**

Current wheeze or use of asthma medication	0.5 (0.1-2.1)	0.8 (0.3-1.8)	**1.8 (1.1-2.8)**	0.5 (0.2-1.5)	0.3 (0.1-1.3)	0.7 (0.4-1.4)	**1.9 (1.2-3.0)**

Reference group: general population (OR = 1)*

OR: Odds Ratio

* adjusted for age, sex, level of education and smoking

Analysis based on grouping by endurance levels showed comparable results: those with a very high endurance level had a statistically significantly increased OR for doctor's diagnosed asthma (2.5; 1.5-4.0), current use of asthma medication (1.9; 1.0-3.5) and current wheeze or use of asthma medication (1.9 1.2-3.0). As for type of sport, no associations between level of endurance and symptoms were seen (table 3).

## Discussion

This study is the first to report results about asthma, allergies and medical treatment in different groups of German top athletes compared to the general population. We found a higher prevalence of doctor's diagnosed asthma in athletes than in the general German population. In addition, we found the highest prevalence of doctor's diagnosed asthma, current use of asthma medication and current wheeze or use of asthma medication in athletes performing in endurance sports. Furthermore, those athletes with a very high endurance level also had a higher prevalence of the three outcomes than the general population.

Seventeen percent of the German top athletes reported a doctor diagnosis of asthma. Similar results were observed in Denmark (16%), Canada (15%), Italy (15%) and in US Summer Olympic athletes where 15% reported doctor's diagnosed asthma [2-4,11]. However, the prevalence was lower than in the UK (21%), Finland (23%) or Australia (26%) [12,15,17]. The prevalence of allergic rhinitis in our study (25%) was quite similar to those in Finnish swimmers (29%) [5] and in Canadian athletes (21%) [4], but higher than in two studies from Switzerland (17%[1]) and Italy (15%[3]). In addition, the prevalence of wheezing in our study (12%) was similar to results of an US study (10% [2]), but two-times higher in comparison to the results of a Norwegian and an Italian study where only 6% of the athletes reported wheezing [3,6]. Ten percent of the participating athletes reported current use of an asthma medication. This result was in accordance with the majority of other studies that also investigated the use of asthma medication in athletes [2,4,12,13].

Two previous studies showed that athletes had an increased risk for allergic symptoms such as allergic rhinitis in comparison to non-athletes [15,28]. We could not confirm these findings. However, as in previous studies [3,13-16], prevalence of asthma diagnosis was higher in top athletes than in the general population. In general, this might be due to a better medical surveillance of the athletes. Another explanation for the lower prevalence in the general population might be that the participants lived mainly at the countryside and it is known that the prevalence of asthma and allergies is lower in these people [26]. Furthermore, it has to be kept in mind that the survey was done 5 years prior to the AQUA-survey, however, it has been recently shown that prevalence of asthma and allergies reached a plateau. As we had to rely on self-reported data it might also be that the reported respiratory symptoms of the athletes during and/or after exercise might be misinterpreted as asthma and could be just exercise-related symptoms.

Athletes performing in endurance sports and those with a very high endurance level also had a significantly increased risk for current use of asthma medication (1.8; 1.0-3.4) and current wheezing or use of asthma medication (1.8; 1.1-2.8). Athletes of the remaining sport types did not differ from to the general population. An explanation for a higher prevalence of asthma in athletes performing endurance sports and those with a very high endurance level might be that these athletes expose their airways to allergens and irritants and their ventilation rate is increased for a longer period of time [19,20,29]. The results indicate that those athletes who have the highest risk for an asthma diagnosis are well monitored by the clinicians and are treated sufficiently against asthma. The control of asthma and the appropriate medical treatment is indispensable to avoid a reduction of the performance of the athletes. However, no dose-response relationship between endurance level and respiratory health was found.

One strength of this study was that we were able to divide the athletes into different groups regarding their endurance levels. This is of importance considering that within the different sports discipline groups the athletes are showing up with relevant differences in endurance levels. However, we were not able to assess possible risks of single sport groups e.g. swimmers or soccer players because the numbers in these groups were too small.

A limitation of our study was the data collection using a self-administered questionnaire. Thus, objective clinical data are missing to underline the observed results. However, validated questions were used and the chance of over reporting is limited as the prevalence of symptoms was not increased. The response of 65% was moderate but comparable to many other populations-based studies.

## Conclusions

Taking socio-demographic differences and smoking habits between top athletes and the general population into account, our study suggest that medical surveillance and treatment in Germany especially in top athletes involved in endurance sports is better than in the general population.

## Abbreviations

AQUA: Allergy Questionnaire for Athletes; ECHRS: European Community Respiratory Health Survey; GA^2^LEN: Global Asthma and Allergy European Networks; TUE: therapeutic use exemption

## Competing interests

The authors declare that they have no competing interests.

## Authors' contributions

ST performed parts of the statistical analysis and drafted the manuscript. BW conceived of the study, and participated in its design and coordination. CW participated in the coordination of the field phase. DN made contributions to draft the manuscript. KR made contributions to conception and design and also to analysis and drafting the manuscript. All authors read and approved the final manuscript.
